# Reduction of HIP2 expression causes motor function impairment and increased vulnerability to dopaminergic degeneration in Parkinson’s disease models

**DOI:** 10.1038/s41419-018-1066-z

**Published:** 2018-10-03

**Authors:** Jinlin Su, Pei Huang, Meiling Qin, Qingqing Lu, Xiao Sang, Yijun Cai, Ying Wang, Fubing Liu, Rong Wu, Xiaoping Wang, Xiaoxing Jiang, Jian Wang, Qiang Sun, Shengdi Chen, Jin Xu

**Affiliations:** 1 Institute of Neuroscience and State Key Laboratory of Neuroscience, CAS Key Laboratory of Primate Neurobiology, Chinese Academy of Sciences, Shanghai, 200031 China; 2 College of Life Sciences, University of Chinese Academy of Sciences, Beijing, 100049 China; 3 Department of Neurology & Institute of Neurology, Ruijin Hospital, Shanghai Jiaotong University School of Medicine, Shanghai, 200025 China; 4 CAS Center for Excellence in Brain Science and Intelligence Technology, Chinese Academy of Sciences, Shanghai, 200031 China; 5 Department of Neurology, Huashan Hospital, Fudan University, Shanghai, 200040 China; 6 Department of Orthopedic Surgery, Zhongshan Hospital, Fudan University, Shanghai, 200032 China; 7 Department of Neurology, Shanghai First People’s Hospital, Shanghai Jiao Tong University School of Medicine, Shanghai, 200080 China

## Abstract

Huntingtin interaction protein 2 (HIP2) is an E2 ubiquitin-conjugating enzyme associated with neurodegenerative diseases, and HIP2 mRNA has been implicated as a potential blood biomarker for Parkinson’s disease (PD). However, it is unclear whether the alteration of HIP2 expression may contribute to the development of PD, and whether the change of HIP2 in blood could reflect its expression in the brain or motor functions in PD patients. In this study, we established a mouse line with HIP2 haploinsufficiency. The reduction of the HIP2 expression led to spontaneous motor function impairment and dopaminergic neuronal loss. Furthermore, HIP2 haploinsufficiency increased the susceptibility of mice to 6-hydroxydopamine (6-OHDA) and caused severe loss of dopaminergic neurons. Interestingly, in a 1-methyl-4-phenyl-1,2,3,6-tetrahydropyridine (MPTP) mouse model for PD, we observed concurrent, highly correlated decrease of HIP2 expression in the brain and in the blood. Using blood samples from more than 300 patients, we validated the decreased HIP2 mRNA in PD patients, including de novo patients. Finally, in a 1-year, 20-patient study, we observed reversed blood HIP2 mRNA levels accompanying improved motor and overall daily functions in 75% of the PD patients with instructed Tai Chi training. Therefore, our in vivo studies have indicated HIP2 insufficiency as a contributing factor for PD, and functionally validated blood HIP2 as a useful and reversible biomarker for PD.

## Introduction

Huntingtin interaction protein 2 (HIP2), also known as UBE2K or E2-25K, was identified as an E2 ubiquitin-conjugating enzyme in the ubiquitin proteasome (UPS) pathway^1^. Besides biochemical interaction^2^, the association between HIP2 and neurodegeneration was strengthened by many subsequent studies. In a cellular model of HD, knockdown of HIP2 reduced huntingtin aggregation formation^3^. However, knockdown of HIP2 homolog in *Caenorhabditis elegans* resulted in decreased aggregation number but increased aggregation size^4^. In 2003, HIP2 was reported as a mediator of Aβ toxicity^5^. Knockdown of HIP2 in neuronal cell line specifically protected cell death induced by Aβ via inhibition of caspase-12 expression and activation^6^. Although these studies provided us with some evidence to support the potential roles of HIP2 in various neurodegenerative diseases, the roles of HIP2 in neurodegeneration in vivo have not been well characterized.

Interestingly, several lines of evidence have suggested that HIP2 could be a potential biomarker for Parkinson’s disease (PD). Decreased expression of HIP2 was found in the substantia nigra (SN) tissues^7^ or in micro-dissected midbrain dopaminergic neurons from post-mortem brains of PD patients^8,9^. Furthermore, the mRNA level was specifically down-regulated in the peripheral blood of PD patients^10–12^. Nevertheless, in another study, HIP2 mRNA was found to be increased in the blood of PD patients^13^. Although sufficient evidence has validated the association between HIP2 and PD, it is unclear how the change of HIP2 expression may contribute to the development of PD, and whether the change of HIP2 in blood could reflect its expression in the brain. It is also unclear whether the HIP2 expression in the blood could change with therapeutic intervention.

To address these questions, we first generated HIP2 haploinsufficient mice using CRISPR-Cas9 gene editing, and characterized their motor functions and pathologic features in the 6-hydroxydopamine (6-OHDA) toxicity model for PD. Next, we evaluated the concurrent change of HIP2 expression in various brain regions and blood using the 1-methyl-4-phenyl-1,2,3,6-tetrahydropyridine (MPTP) model for PD. Finally, we monitored and compared the changes in clinical features and blood HIP2 expression in a group of 20 PD patients with supervised Tai Chi training. Our results indicated that decreased HIP2 expression increased the dopaminergic vulnerability in the 6-OHDA model for PD, and the reduced blood HIP2 faithfully reflected the similar changes in the brain. Furthermore, beneficial non-medical intervention, such as Tai Chi, was able to reverse the blood HIP2 expression. Therefore, our in vivo studies have indicated HIP2 insufficiency as a contributing factor for PD, and the low blood level of HIP2 as a useful PD biomarker.

## Results

### Generation of HIP2 knockout mice

**Fig. 1:**
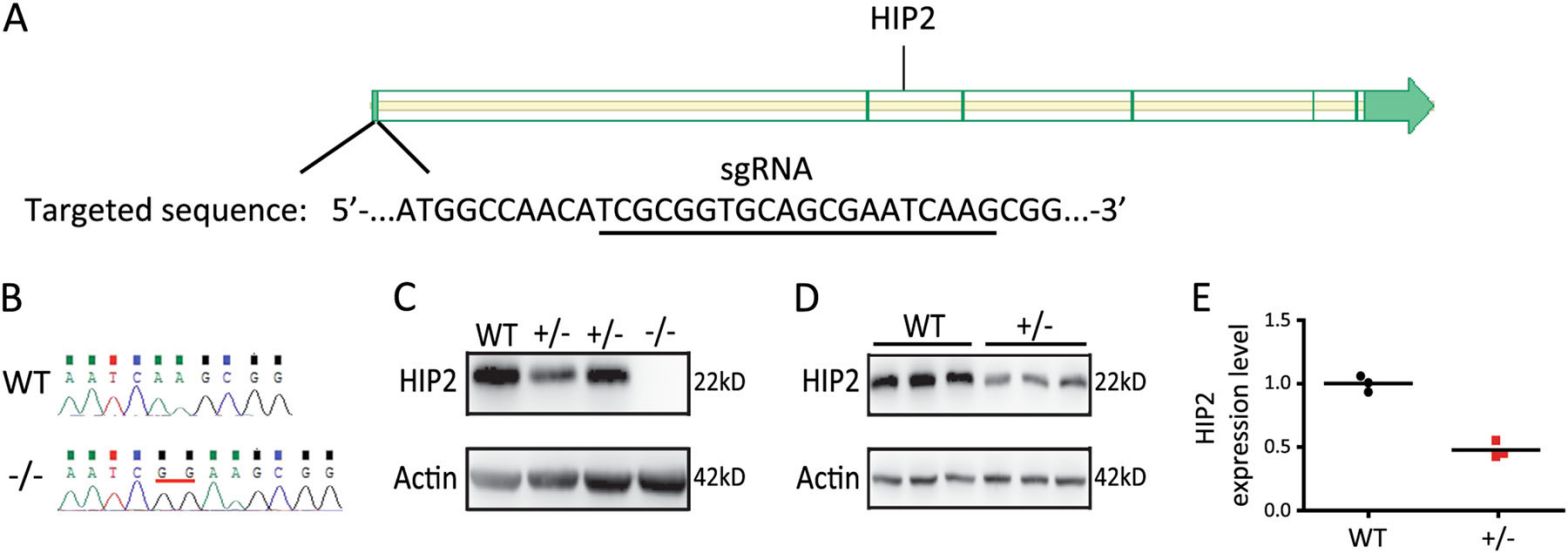
Generation and verification of HIP2 KO mice. **a** Schematic representation of sgRNA-targeting sequence of HIP2. **b** Sequencing result of WT and HIP2 KO (HIP2^−/−^) embryonic fibroblasts. **c** HIP2 expression was decreased in HIP2^+/−^ fibroblast and eliminated in HIP2^−/−^ cell. **d** Immunoblots showing reduced HIP2 expression in the cortex of HIP2^+/−^ mice, and quantification is presented in (**e**), (*P* = 0.0006 by unpaired *t*-test)

To investigate the role of HIP2 in vivo, we generated HIP2-deficient mice using CRISPR-Cas9 system. The sgRNA targeted the N-terminal region of HIP2 (Fig. 1a) and the gene editing caused a frame-shift and early termination (Supplementary S1). We did not detect off target effects at predicted sites (Supplementary S1D), and potential consequences derived from undetected off target effects were minimized by multiple rounds of breeding with the wild-type animals. Only one line (#27) generated offspring beyond F1 (Supplementary S1C), and this line (HIP2 KO) was used in breeding for all the experiments in subsequent studies. Among neonatal mice generated from breeding pairs between 2 heterozygous HIP2 KO (HIP2^+/−^), 37.5% of them were wildtype (WT) while 62.5% of them were heterozygotes. No homozygous gene-edited knockout (HIP2^−/−^) mouse was found at birth, suggesting homozygotes to be embryonic lethal. By examining 14-day old embryos, we found about 20% of all embryos were HIP2^−/−^, verified by sequencing of PCR product (Fig. 1b). Immunoblotting result showed a clear decrease of HIP2 expression in heterozygotes (HIP2^+/−^) embryos and total vanish of HIP2 in homozygote embryos (Fig. 1c). We also found a significant decrease of HIP2 expression in the cortex of adult heterozygotes (Fig. 1d, e). Taking together, we have generated a mouse model with HIP2 haploinsufficiency.

### Motor deficits in adult HIP2^+/−^ mice

**Fig. 2:**
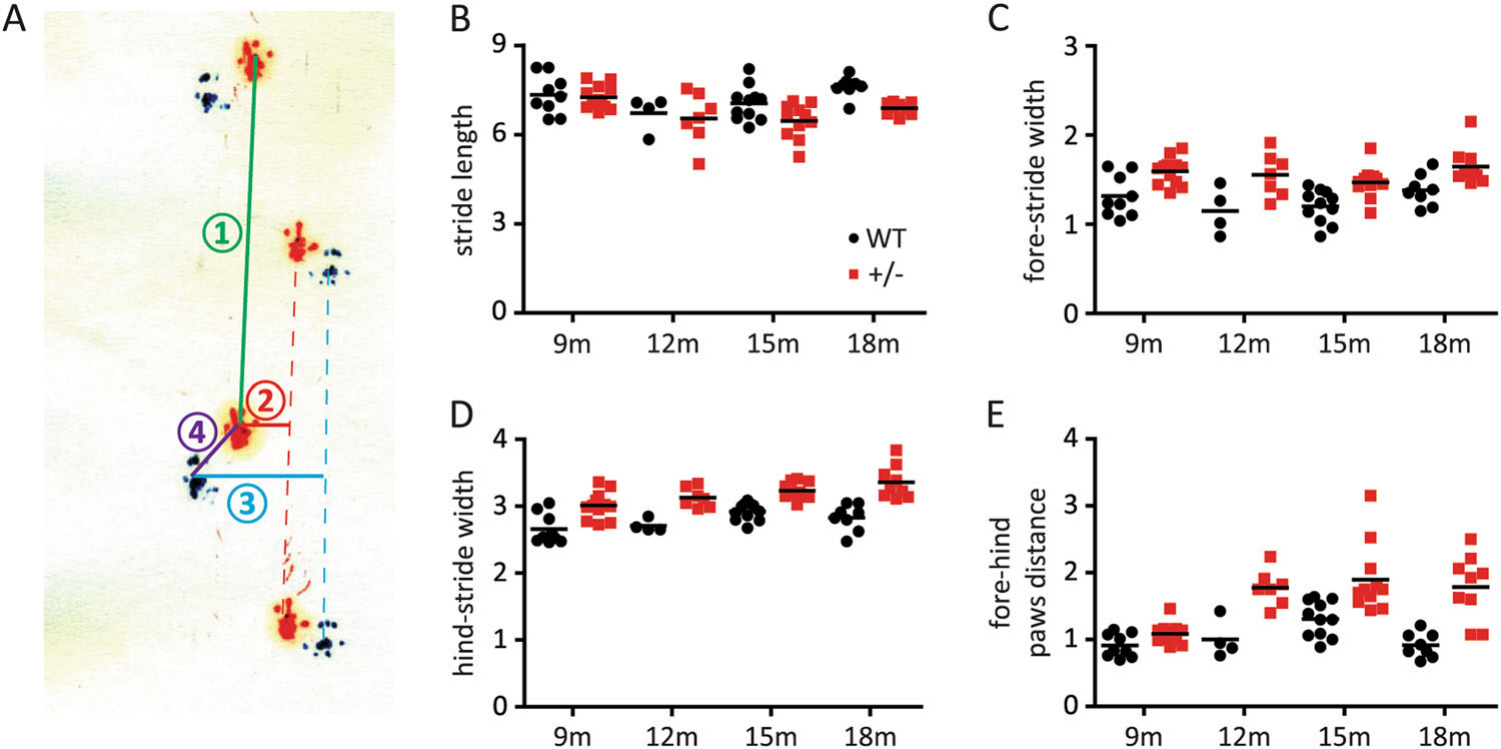
Spontaneous gait abnormality of HIP2^+/−^ mice. **a** Schematic representation of the definition for stride length ①, fore-stride width ②, hind-stride width ③, and forepaw–hindpaw distance ④ measurement. The details are described in “Materials and methods”. **b** HIP2^+/−^ mice showed significant shorter distance in stride length at the age of 15 months (*P* = 0.0272) and 18 months (*P* = 0.0001). **c**, **d** HIP2^+/−^ mice had broader fore-stride width (**c**, *P* values for mice with indicated ages are: 0.0038 (9 months), 0.0297 (12 months), 0.0023 (15 months), 0.014 (18 months)) and hind-stride width (**d**, *P* values are: 0.0012 (9 months), 0.0006 (12 months), <0.0001 (15 months), 0.0002 (18 months)) at all ages than WT mice. **e** HIP2^+/−^ mice showed significant difference in fore–hind paws distance at all ages (*P* values: 0.0257 (9 months), 0.0016 (12 months), 0.0031 (15 months), 0.0003 (18 months)). The number of animals in each group is listed in “Materials and methods”. All *P* values in this figure were analyzed by unpaired *t*-test

Given the association of HIP2 to PD, we evaluated the motor functions of HIP2^+/−^ mice. We first examined potential gait defects of mice by using footprint analysis. We chose stride length, fore-/hind-stride width representing gait stability, and forepaw–hindpaw distance representing gait uniformity as main parameters for analysis (Fig. 2a). Normally, mice walk in a straight line with a steady gait and place their hindpaws where forepaws touch. In our study, we observed shortened stride length in 15-month-old and 18-month-old HIP2^+/−^ mice (Fig. 2b). Furthermore, we found that HIP2^+/−^ mice had significantly increased fore-stride width and hind-stride width from 9-month and above when compared to WT mice, suggesting they need a bigger area to maintain balance (Fig. 2c, d). HIP2^+/−^ mice also had significantly longer forepaw–hindpaw distance, indicating that they had an irregular walking pattern with more wobbles and swings (Fig. 2e).

**Fig. 3:**
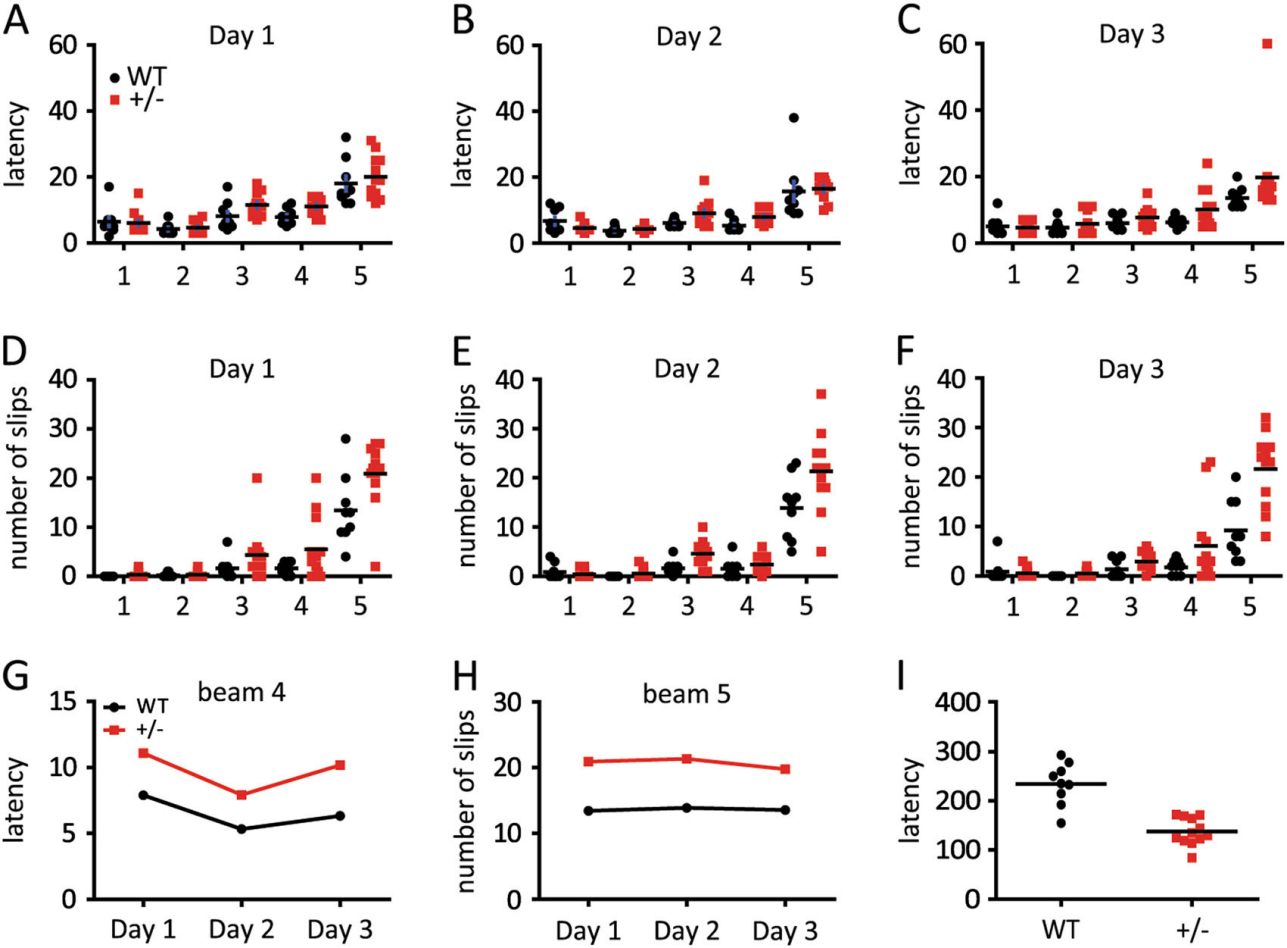
Spontaneous motor deficits occurred in HIP2^+/−^ mice at 9-month age. **a**–**c** The latency of mice crossing beams 1–5 of narrowing width in beam walking test. On days 1 (**a**, *P* = 0.0307) and 3 (**c**, *P* = 0.0179), there were statistically significant difference in the latency between the WT and HIP2^+/−^ mice. **d**–**f** The number of slip-offs from the beams for mice crossing the beams. Note that the HIP2^+/−^ mice slipped more frequently when crossing beams in all 3 days (**d**, *P* = 0.0009; **e**, *P* = 0.0017; **f**, *P* < 0.0001). **g**, **h** HIP2^+/−^ mice showed longer latency for crossing beam 4 (*P* = 0.0003) and more slips crossing beam 5 (*P* = 0.0108) in all 3 days. The gradually increased latency and number of slip-offs for mice on different beams were caused by different task difficulties (*P* < 0.0001 in all 3 days). All *P* values of beam walking test results were analyzed by two-way ANOVA followed by Bonferroni’s post-hoc test. **i** Rotarod test showing the shorter latency for HIP2^+/−^ mice before falling off than for the WT mice (*P* < 0.0001 by unpaired *t*-test). *N* = 9 mice for WT and 12 mice for HIP2^+/−^

We then used beam walk test to further characterize the balance of HIP2^+/−^ mice. Five beams with different shapes and diameters were used to create different task difficulties. The widest one was designated as beam 1 and the narrowest one as beam 5. Normally, the latency and number of slip-off during beam crossing rise from beam 1 to beam 5 (Fig. 3a–f). In our study, all mice traversed beams 1 and 2 with ease during 3 test days, showing no difference between the WT and HIP2^+/−^ mice. However, for narrower beam 3, HIP2^+/−^ mice started to need longer time to cross and showed more slip-offs (Fig. 3a–f). For beam 4, on each of the 3 test days, the HIP2^+/−^ mice required more time to cross the beam (Fig. 3g) and had more slips (Fig. 3a–f). Although the latency for the WT and HIP2^+/−^ mice to cross the narrowest beam 5 was not much different for the first 2 days likely due to significantly increased difficulty, on 3rd day the WT mice showed reduced latency compared to the HIP2^+/−^ mice (Fig. 3a–c). Furthermore, HIP2^+/−^ mice made about 50% more slips than WT mice in all 3 days test (Fig. 3h). Finally, we performed rotarod test and found that HIP2^+/−^ mice showed much shorter duration on rotating rod (Fig. 3i). Taken together, these results clearly demonstrated that HIP2^+/−^ mice had motor deficits.

### Increased susceptibility of HIP2^+/−^ mice to 6-OHDA toxicity

Since reduced HIP2 expression could cause motor deficits in mice and has been associated with PD patients^7–12^, we hypothesized that decrease of HIP2 may cause increased susceptibility to toxins commonly used to generate mouse models of PD. To test this idea, we adopted the unilateral 6-OHDA lesion model^14,15^ mimicking pathological features of PD. In this model, 6-OHDA was injected into one side of medial forebrain bundle (MFB) and saline was injected into the contralateral side as control. As a result, each experimental mouse suffered depletion of nigro-striatal tract on 6-OHDA-injected side of the brain while neurons and projections remained intact on saline-injected side, thus enabling us to quantify neuronal damage in the same mouse. Meanwhile, apomorphine could induce asymmetric rotation on all experimental mice due to hypersensitivity in lesioned striatum, and allowed us to assess brain lesion via rotating behavior.

**Fig. 4:**
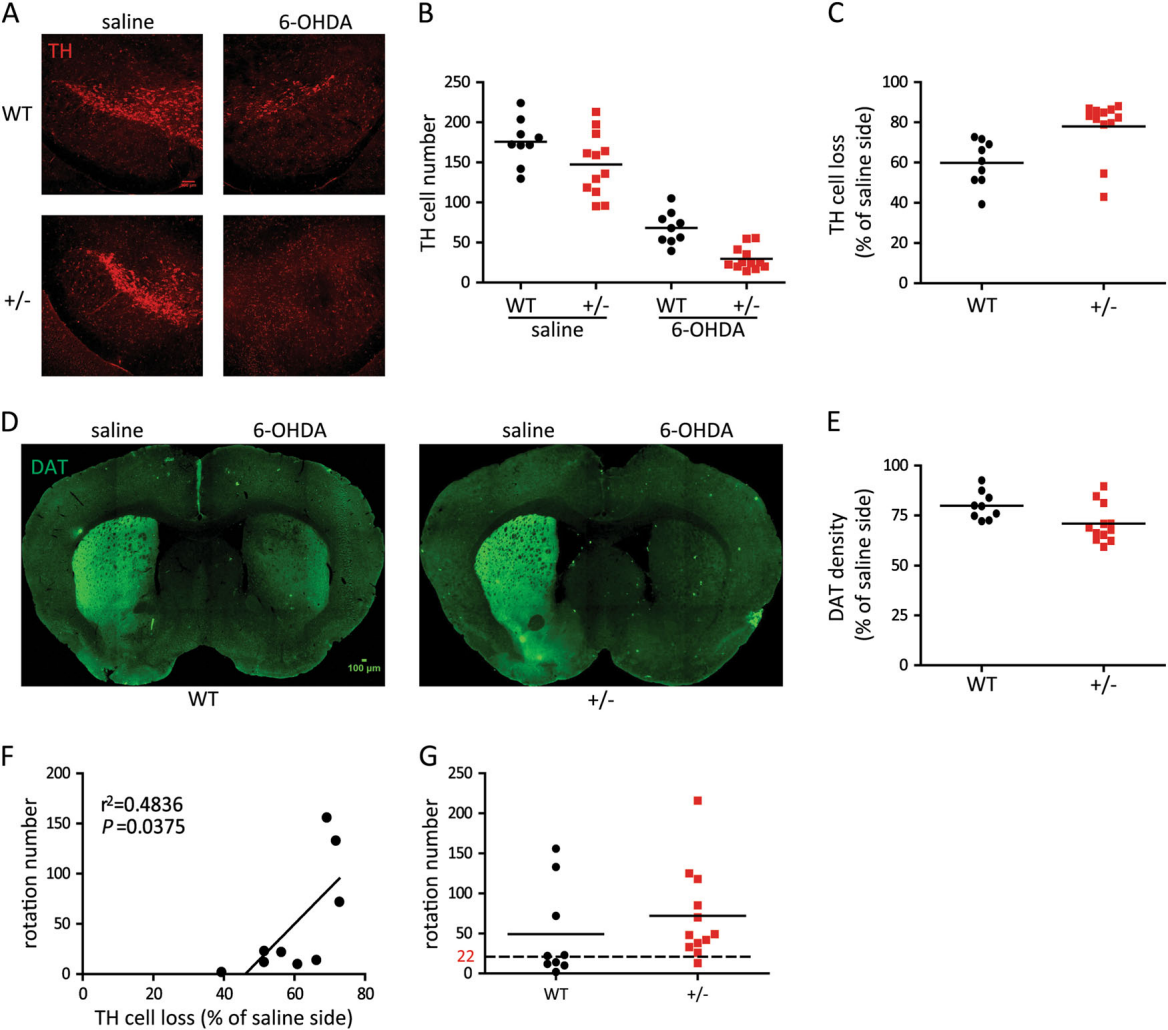
6-OHDA caused more severe pathological and behavior damages in HIP2^+/−^ mice. **a**–**c** Immuno-fluorescence staining of remaining dopaminergic neurons in the SN after saline and 6-OHDA injection in the WT or HIP2^+/−^ mice. The number of TH^+^ cells in the WT or HIP2^+/−^ mice after injection are shown in (**b**). 6-OHDA injection significantly decreased the number of remaining TH^+^ cells in both WT and HIP2^+/−^ mice (*P* < 0.0001 by two-way ANOVA) and that of HIP2^+/−^ mice was more severe (*P* = 0.0056 by Bonferroni’s post-hoc test). In saline-injected side, TH^+^ cells number difference between WT and HIP2^+/−^ mice was not significant (*P* = 0.0504 by Bonferroni’s post-hoc test). The relative loss of TH cells in the WT or HIP2^+/−^ mice after 6-OHDA injection when compared to the saline-injected side was plotted in (**c**, *P* = 0.0053 by unpaired *t*-test). **d** Immuno-fluorescence staining of striatal dopaminergic projection in the WT or HIP2^+/−^ mice injected with saline and 6-OHDA, with quantification of the signals in (**e**, *P* = 0.0263 by unpaired *t*-test). **f** Correlation of apomorphine-induced rotation and dopaminergic neuron loss level in WT mice (*R*^2^ = 0.4836, *P* = 0.0375 by Pearson’s coefficient). **g** Increased apomorphine-induced rotation in HIP2^+/−^ mice. Dashed line denotes the median number of rotations (22) in the WT group. Note 11 out of 12 HIP2^+/−^ mice showed higher number of rotation than this value. Scale bar = 100 μm. *N* = 9 mice for WT and 12 mice for HIP2^+/−^

In our study, 6-OHDA induced degeneration of dopaminergic neurons in SN and denervation in striatum as expected. Interestingly, in the saline-injected side, HIP2^+/−^ mice had fewer TH^+^ neurons than WT mice (Fig. 4a), indicating HIP2 insufficiency may affect the survival of dopaminergic neurons even without external toxin. In the lesion side, HIP2^+/−^ mice suffered more significant loss of TH^+^ neurons (Fig. 4a–c) and more severe loss of striatal dopamine transporter (DAT) labeling projection (Fig. 4d, e), indicating increased susceptibility of HIP2^+/−^ mice to 6-OHDA. It is worth noting that in the WT mice, injection of 6-OHDA also led to decreased expression of endogenous HIP2 in SN and striatum, but not in cortex and hippocampus (Supplementary S2B, E).

Apomorphine-induced rotations were analyzed before the mice were sacrificed (Supplementary S2F) so the correlation of behavioral and pathological changes could be assessed. For the 9 WT mice, there was certain degree of variation in the dopaminergic neuronal loss (Fig. 4c), but the correlation between the number of apomorphine-induced rotations and the loss of TH^+^ neurons was strong (*R*^2^ = 0.4836, *P* = 0.0375, Fig. 4f). To examine whether increased dopaminergic neuronal loss in HIP2^+/−^ mice was also reflected in rotation test, we compared the number of rotations of these mice. The median value of the rotations for WT mice was 22 rounds in 10 min. For HIP2^+/−^ mice, 11 out of 12 mice showed higher number of rotations than 22 (Fig. 4g). Therefore, consistent with increased loss of dopaminergic neurons, the behavioral changes also demonstrated increased susceptibility of HIP2^+/−^ mice to 6-OHDA. We have also attempted to validate increased susceptibility of HIP2^+/−^ mice using the MPTP model. However, the higher toxicity of MPTP has caused significant death among experimental groups and prevented us from performing reliable analysis.

### Concurrent reduction of HIP2 mRNA in the blood and brain of MPTP mice

Although there is evidence supporting the reduction of HIP2 expression in the post-mortem brains and in peripheral blood of PD patients^7–12^, whether these events occur simultaneously and whether the change in blood HIP2 reflects a similar change in the brain are unknown due to the impossibility for this study in patients. To answer these questions using rodent model, we adopted the acute MPTP mouse model in WT mice. MPTP is a toxin targeting mitochondrial complex I and is commonly used to establish animal models of PD^16^. Compared to 6-OHDA, MPTP selectively extinguishes dopaminergic neurons without affecting the survival of adrenergic neurons. Intraperitoneal injection of MPTP induces not only bilateral lesion of nigro-striatal tract but also generates other PD-associated features, including inflammatory response^17^ and extracellular glutamate elevation^18^, thus representing a more comprehensive pharmacological model of PD.

**Fig. 5:**
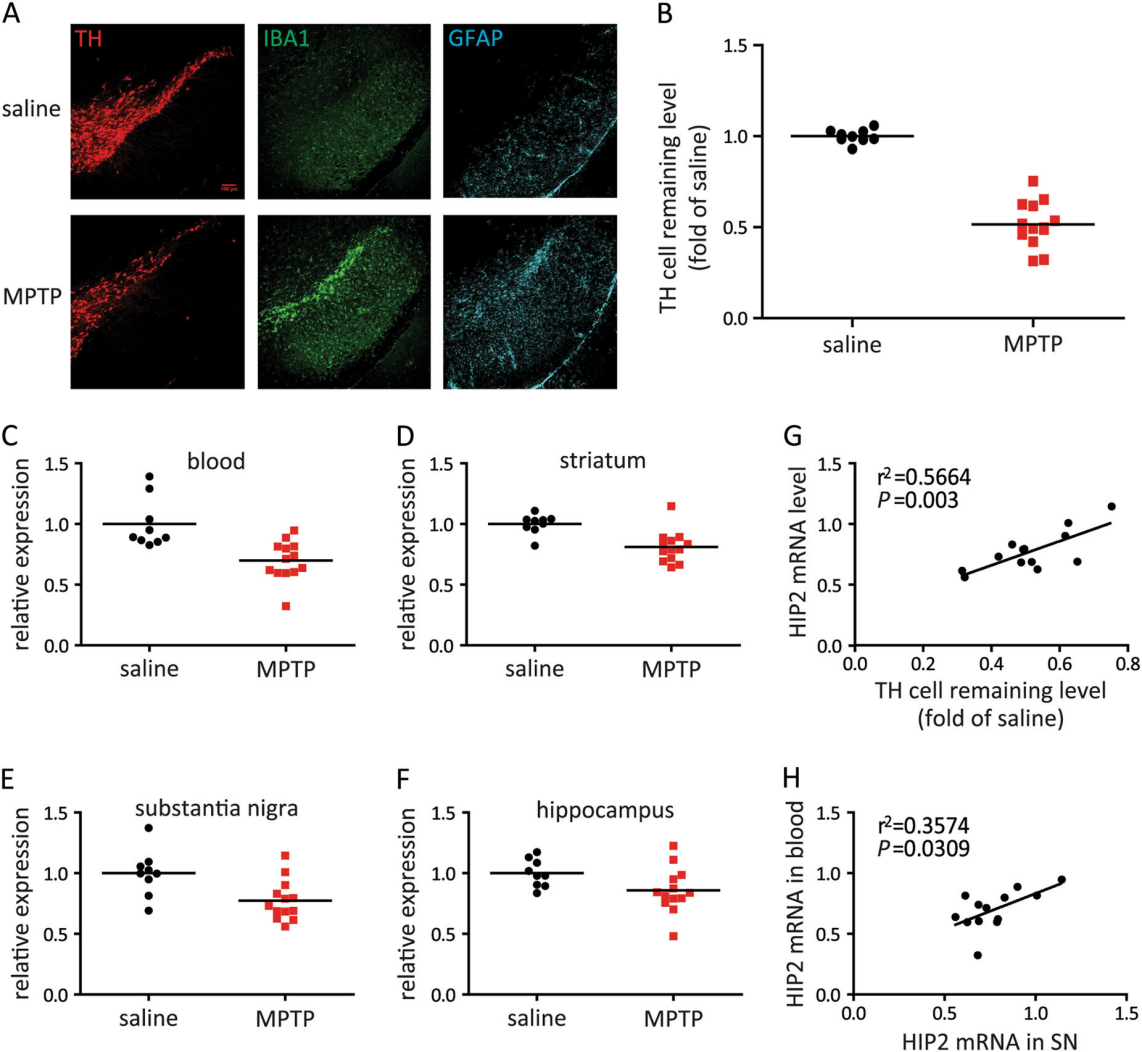
HIP2 mRNA level is decreased in both blood and brain of MPTP-treated mice. **a** Immuno-fluorescence staining of dopaminergic neurons (TH), microglia (Iba1), and astrocytes (GFAP) in the SN of mice injected with saline or MPTP. **b** Relative survival rate of dopaminergic neurons in mice injected with saline or MPTP. MPTP-treated mice showed more than 50% decrease of TH^+^ neurons (*P* < 0.0001). **c**–**f** The HIP2 mRNA levels in the blood (**c**, *P* = 0.001), striatum (**d**, *P* = 0.0009), substantia nigra (**e**, *P* = 0.0075) or hippocampus (**f**, *P* = 0.05) of saline or MPTP-injected mice. All *P* values stated in (**b**–**f**) were analyzed by unpaired *t*-test. **g** The correlation between the survival rate of dopaminergic neurons and HIP2 mRNA level in the SN of MPTP-injected mice (*R*^2^ = 0.6630, *P* = 0.0007 by Pearson’s coefficient). **h** Correlation of the HIP2 mRNA levels in the SN and blood of MPTP-injected mice (*R*^2^ = 0.3574, *P* = 0.0309 by Pearson’s coefficient). Scale bar = 100 μm. *N* = 9 in saline group and *n* = 13 in MPTP group

The efficacy of MPTP in our experiments was validated by observed 50% dopaminergic neuronal loss and the accompanying gliosis of astrocyte and microglia (Fig. 5a, b). We collected the samples 7 days after MPTP injection, and detected a decrease of HIP2 mRNA in both blood and brain (Fig. 5c–f). Interestingly, the decrease of HIP2 in the striatum and SN were more significant than that in the hippocampus, consistent with the observation from 6-OHDA-injected mice (Supplementary S2B–E). Linear regression analysis revealed a high degree of correlation between the number of surviving dopaminergic cells and HIP2 mRNA level in SN (*R*^2^ = 0.5664, *P* = 0.003, Fig. 5g). Moreover, the correlation between the HIP2 level in SN and blood was also statistically significant (*R*^2^ = 0.3574, *P* = 0.0309, Fig. 5h). Taken together, our analysis indicates that in the MPTP mouse model, the reduced level of HIP2 mRNA reflects the concurrent change in the brain, especially in the brain regions that are affected by PD.

### Reversal of blood HIP2 mRNA expression in PD patients after Tai Chi exercises

Together with increased vulnerability of dopaminergic neurons observed in mice with reduced HIP2 expression, the concurrent reduction of HIP2 expression in the blood and brain in the MPTP model strengthened the idea that HIP2 expression in blood could be a useful and convenient biomarker for PD. To help to resolve the conflicting results on the change of HIP2 expression in blood^10–13^, we collected blood samples from 211 PD patients and 91 age-matched healthy controls, and measured the HIP2 mRNA from whole blood (Supplementary S3, 4). Consistent with prior studies^10–12^, our results showed decreased HIP2 mRNA expression in PD patients (Supplementary S3A, B). Furthermore, by comparing PD patients with (medication group) or without (de novo group) medicine treatment, we found that HIP2 mRNA level was decreased in de novo PD patients and that the HIP2 expression was not affected by levodopa-related medical treatment (Supplementary S3C, D).

Previous studies suggested some exercises, including treadmill, tango, and Tai Chi, could ameliorate some motor deficits in PD patients^19–22^. These non-medical interventions may complement levodopa-based therapy to further improve the wellness of PD patients. In a well-designed and controlled study, Li et al. have demonstrated that twice-a-week instructed Tai Chi could improve the balance and other motor functions in PD patients 6 months after training^23^. Using the same protocol, we enrolled 20 stage I–III PD patients (Supplementary S5) into a 1-year pilot program to assess whether Tai Chi exercises could affect HIP2 mRNA level in blood and how the changes could be correlated with the patients’ overall mental and physical performance.

**Fig. 6:**
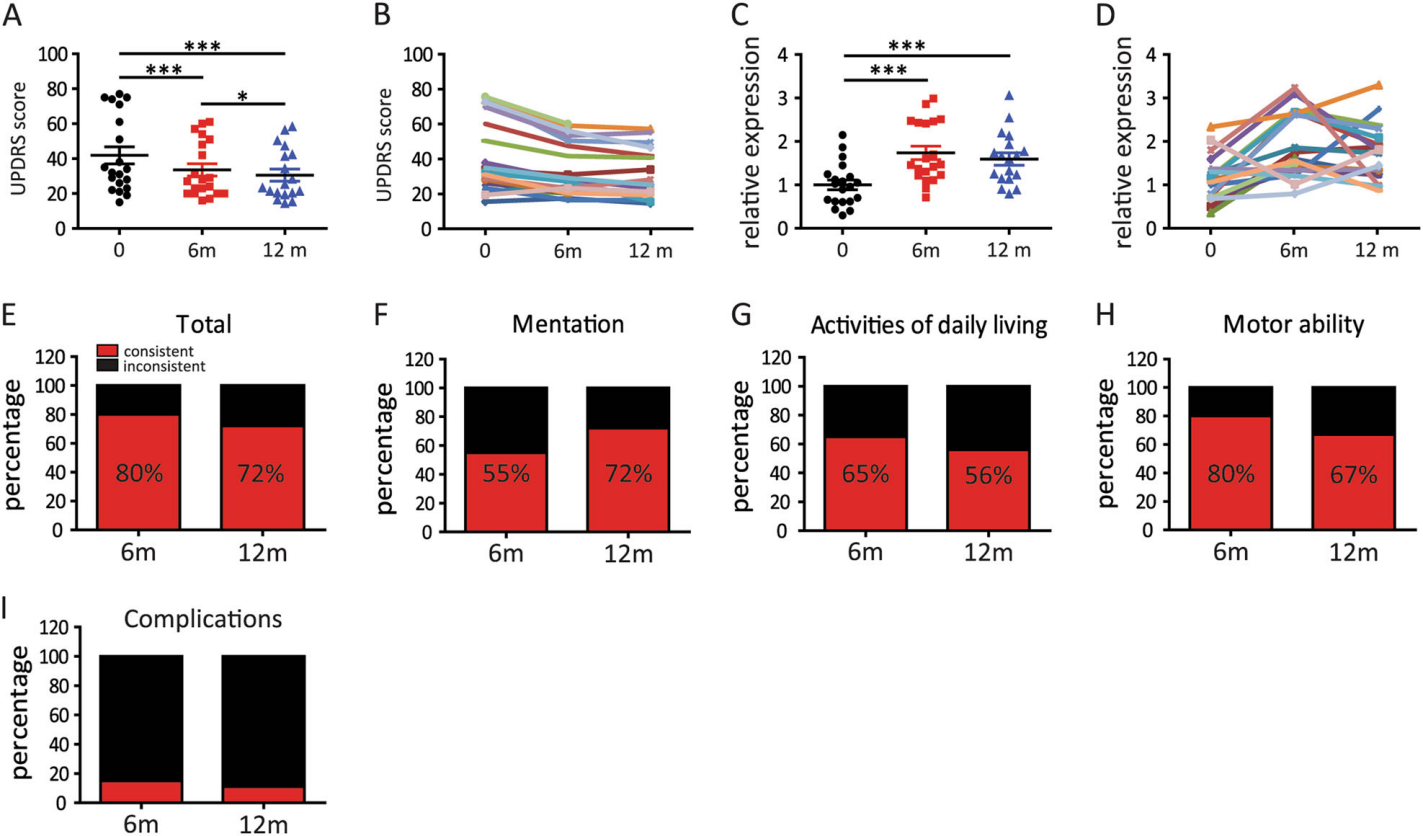
The HIP2 mRNA is increased in the blood of PD patients after Tai Chi training. **a** Decreased UPDRS-motor scores in PD patients with 6 and 12 months of Tai Chi exercises. **b** The change of UPDRS-motor score of each patient. **c** Reversal of the HIP2 mRNA level in the blood of patients with Tai Chi exercises. **d** The change of HIP2 mRNA level in each patient. **a**–**d** Detailed demographic information was listed in Supplementary S4. **e**–**i** The consistency of the change of HIP2 level with the change of behaviors in clinical assessment in PD patients undergoing Tai Chi exercises. Data are analyzed by paired *t*-test and presented as mean ± SEM. *P < 0.05, ***P < 0.001

In our study, all the patients completed the 6-month instructed Tai Chi exercises, and 17 of the 20 patients (85%) showed decreased overall Universal Parkinson’s Disease Rating Scales (UPDRS) scores, suggesting an improvement by clinical standards (Fig. 6a, b). Among those 17 patients, 15 of them showed increased HIP2 expression (Fig. 6c, d). One patient exhibited decreased HIP2 expression and increased UPDRS score, suggesting the lack of improvement determined by both the clinical assessment and HIP2 level. Therefore, 80% of the test subjects showed consistent change between the clinical outcome and HIP2 levels (Fig. 6e). Two patients dropped out of the study by the end of 12-month training. For the remaining patients, 16 of them (89%) achieved improvement by clinical UPDRS standards, with most of them showing increased HIP2 levels when compared to those at the beginning of the study (Fig. 6c). The consistency between the clinical assessment and the direction of HIP2 change was 72% at 12-month time point, even with only 18 patients remaining (Fig. 6e). It is worth noting that significant clinical improvement and the increased HIP2 expression level occurred after just first 6-months of Tai Chi exercises.

As UPDRS contains a variety of categories including mental status, daily activities, motor functions, and therapy complications, we performed a subgroup analysis to further investigate the effect of Tai Chi and the correlation between the change of HIP2 expression and patient behaviors (Fig. 6e–i, Supplementary S3E–I). Consistent with previous studies, our results indicated clear benefits of Tai Chi exercises for the motor function improvement (UPDRS III), and a noted improvement in mental and mood status (UPDRS I) and daily tasks performance (UPDRS II). Among these categories, motor function improvement has the highest correlation with increased HIP2 expression, suggesting increased HIP2 levels may be a molecular indicator for motor functions in PD patients.

## Discussion

In this study, by using various in vivo models, we have demonstrated that reduced HIP2 expression contributed to dopaminergic neuronal loss and motor deficits. Furthermore, we have shown that blood HIP2 expression could reflect the state in the brain and could be a useful biomarker for motor functions in PD patients. Therefore, our results have generated novel insights into the functional relevance of a potential PD biomarker.

A plethora of peripherally-based biomarkers have been proposed for PD using data generated from transcriptome and proteomic studies^24–26^. Nevertheless, for most if not all of them, follow-up functional studies are absent to assess whether the peripheral changes observed would correspond to changes in the brain in PD models. Thus, the lack of understanding of the disease-relevance of those potential biomarkers has limited their application in the clinic. HIP2 was also identified via transcriptome analysis of blood samples from PD patients as one of the molecular signatures for PD^10^. Given that decreased HIP2 expression has been reported in the blood and SN tissues from PD patients, and that HIP2 is an E2 conjugating enzyme in the UPS pathway known to be affected in PD^27^, HIP2 could be a genuine disease-relevant PD biomarker. Our current study is the first in vivo analysis of the effects of reduced HIP2 on pathology and motor deficits associated with PD. Our results have indicated that the HIP2 expression in the SN and striatum is reduced in the WT mice exposed to 6-OHDA or MPTP. Furthermore, reduced expression of HIP2 in the brain by genetic approach increased the vulnerability of dopaminergic neurons and leads to motor function deficits in mice, thus suggesting reduced HIP2 expression observed in PD patients is likely a contributing factor for disease progression.

Intriguingly, MPTP could cause a concurrent decrease of HIP2 expression in the brain and in blood. The metabolite of MPTP, MPP^+^, specifically targets the mitochondria complex I of dopaminergic neurons and cause degeneration. It is unclear how MPTP would affect the gene expression patterns in blood. However, MPTP-induced gene expression change in blood appears to be common, and in some cases is consistent with the changes in the brain^28,29^. Given that the concurrent change of HIP2 in blood and brain could not be realistically assessed in PD patients, our study provided the first in vivo evidence to demonstrate such a change in an established model of PD.

By analyzing the expression of blood HIP2 level in newly diagnosed PD patients (de novo) and patients receiving L-dopa based medical treatment, we found medical treatment did not have a significant effect on HIP2 expression. In contrast, Tai Chi exercise, which is known to improve balance and motor functions in PD patients^23,30–33^, could increase the blood level of HIP2. Furthermore, the reversed expression of HIP2 in blood has a stronger correlation with improved motor functions measured in UPDRS III. Therefore, the change of HIP2 may be an indicator of motor functions. As mitochondrial functions are vital for both dopaminergic neuronal survival and muscle activities, the HIP2 expression could be tightly associated with mitochondrial functions.

In summary, our studies clearly demonstrated that decreased HIP2 expression could lead to increased vulnerability to dopaminergic neuronal death and motor function impairment, and indicated HIP2 mRNA as a reversible blood biomarker for motor functions in PD. These studies set the foundation for future investigations to decipher the molecular mechanisms underlying the changed HIP2 expression in PD, and to assess whether HIP2 could be a therapeutic target for PD.

## Materials and methods

### Mice

HIP2 knockout mice were generated using CRISPR-Cas9 system. The efficiency of cleavage caused by various small guide RNAs (sgRNA) was first evaluated. After selecting the sgRNA that has >40% cleavage efficiency, Cas9 plasmids, sgRNA, and repair oligos (at a concentration of 50 + 100 + 100 ng/μl) targeting 27–29 bp of the coding sequence were injected into the embryos (Supplementary S1A). This strategy was adopted to generate 2 different HIP2 mutant lines at the same time. 220 embryos were injected followed by transplantation to 7 surrogate female mice (Supplementary S1B). One line would harbor a point mutation leading to a change of lysine to arginine at residue 10 (K10R), and the other one (HIP2 KO) would harbor early stop codon caused by insertion or deletion during homologous recombination. The detailed analysis is included in Supplementary S1. The mutant line HIP2 K10R was abandoned due to lack of phenotype and not relevant to current study, and only the HIP2 KO line was used in this study. Top eight potential algorithm-predicted off-target effects (http://www.rgenome.net/cas-offinder/) from sgRNA sequences were examined by PCR amplification followed by sequencing, and no unintended gene editing was found. After three generations of breeding with the WT mice to further minimize potential consequence of off-target effects, heterozygous gene-edited mice (HIP2^+/−^) derived from this founder were used for subsequent research.

Genotype detection was determined based on sequencing result of PCR products of mouse HIP2-specific primer (F: 5′-GCGGAGGTGATTCTACAGTGAGGA-3′, R: 5′-CCGCCTAGTCACACTTCCAGATGTT-3′). All mice in this study were on C57BL/6J background and were bred and maintained under the regulations of the Animal Care and Use Committee of the Institute of Neuroscience, Shanghai Institute of Biological Sciences.

### Immunoblot

Samples collected from mice or cell culture were lysed by RIPA buffer containing protease inhibitor cocktail (04693132001, Roche) on ice. Equal amounts of protein were electrophoresed in 12% SDS-PAGE gel and transferred to PVDF membranes. These membranes were incubated with primary antibodies of HIP2 (ab52930, Abcam) and actin (m20010, Abmart) at 4 ℃ overnight, followed by secondary antibodies incubation at room temperature for 2 h. Images were obtained by Amersham (Imager 600, GE) and quantified by Quantity One (version 4.6.2, Bio-Rad).

### Behavioral tests

A total of 71 male mice were divided as WT group (*n* = 32) or HIP2^+/−^ group (*n* = 39) for behavioral tests. Footprint analysis was performed on 4 groups of mice with different ages. 9-month group had 9 WT mice and 12 HIP2^+/−^ mice, 12-month group had 4 WT mice and 7 HIP2^+/−^ mice, 15-month group had 11 WT mice and 11 HIP2^+/−^ mice, and 18-month group had 8 WT mice and 9 HIP2^+/−^ mice. Beam walking test and rotarod test were just performed on 9-month group (9 WT mice and 12 HIP2^+/−^ mice).

#### Footprint analysis

All mice received 2 days of training (3 runs/day) before 1-day test. To obtain footprints, they were trained to walk along a 50 cm-long, 10 cm-wide, and 10 cm-high corridor leading to an enclosed box. Five minutes of habituation before training and test was necessary for reliable result. During test day, mice were painted with blue and red oil-paint to distinguish forepaws and hindpaws. Paper on the floor of the corridor was switched after every run.

Four parameters were measured from 4 consecutive strides for analysis. Stride length was defined as average distance of each forepaw print in the same side. Forepaw width, similar as hindpaw width, was defined as average distance between each left and right side of forepaw or hindpaw footprint line. Forepaw–hindpaw distance, also named as footprint overlap or relative paw placement, was defined as average distance of each fore- and hindpaw print in the same side. The mean value of all 8 results (including both left and right side of 4 strides) was used for subsequent analysis of each parameter.

#### Beam walking test

The apparatus was set up based on previous study^34^ and five beams (28 mm square beam, 25 mm round beam, 12 mm square beam, 10 mm round beam, and 4 mm square beam) were used. All mice received 2 consecutive days training (5 runs/day on the widest beam) and 3 days test (1 run/day on each of 5 beams). Mice were placed on the open end of walking beam and trained to run to an enclosed box. Mice that failed to ran into box after a maximum of 60 s would be removed from beams and those accidentally fell off the walking beam would be placed back to cage for 2 min. Five minutes of habituation before training and test was necessary to obtain reliable result. The latency of each run and the number of hindpaw slipping off walking beam during running were recorded for subsequent analysis.

#### Rotarod test

All mice received 2 days of training (5 trails/day) before 1-day test on ROTA ROD (47600, Ugo Basile). At day 3, mice were tested 4 times under accelerating mode (4–40 rpm in 5 min) and mean latency before falling off was recorded. Any mouse did not fall off after a maximum latency of 300 s would be removed from rod and placed into cage with other mice. The mean value of 3 longest latencies of each mouse was used for subsequent analysis.

### 6-OHDA-induced PD mouse model and apomorphine-induced rotation test

Twenty-one male mice were divided as WT group (*n* = 9) or HIP2^+/−^ group (*n* = 12) for 6-OHDA PD model establishment and rotation test. All mice received stereotaxic injection of 6-OHDA and saline in the brain at day 0 and rotation test at the day 21. Mice were sacrificed for brain samples at the 28th day post operation (Supplementary S1).

The operation protocol was based on previous studies^14, 15^. Briefly, mice were intraperitoneally injected with 25 mg/kg of desipramine hydrochloride (D3900, Sigma-Aldrich) and 25 mg/kg of pargyline hydrochloride (P8013, Sigma-Aldrich) 30 min before surgery. Then they were anesthetized and injected with 1 μg of 6-OHDA (H116, Sigma-Aldrich) into one side of MFB (A/P: −1.2 mm, M/L: −1.1 mm, D/V: −5 mm). After awaking from unconsciousness on heating pad, mice were delivered 1 ml of glucose-saline solution subcutaneously and raised in cage with soaked food pellets and 5% sucrose solution. Daily glucose-saline solution injection was carried on during whole recovery period.

Rotation test was induced by apomorphine (A4393, Sigma-Aldrich). Apomorphine was intraperitoneal injected with the dose of 0.5 mg/kg for each mouse 10 min before recording. Mice were then placed in an opaque cylinder (30 cm diameter) and filmed for 10 min. Ipsilateral and contralateral rotations were counted single-blinded.

### MPTP-induced PD mouse model

A total of 22 eight-week-old wildtype male mice were divided as saline group (*n* = 9) or MPTP group (*n* = 13) and kept in the procedure room at 22 ℃ for 3 days for acclimation before operation. Then they were administered with saline or MPTP (M0896, Sigma-Aldrich) 4 times at 2 h intervals by intraperitoneal injections. The first two injections were applied with a dosage of 22 mg/kg at 22 ℃ and the last two injections were applied with dosage of 18 mg/kg at 24–25 ℃. Mice were sacrificed for blood and brain samples at the 8th day post operation.

### Animal samples collection

Blood samples were collected in BD vacutainer EDTA as soon as mice were anesthetized. After euthanizing, the animals were perfused with 0.1 M PBS (pH 7.4). For samples from 6-OHDA injected animals, mice were perfused with 4% paraformaldehyde (PFA) solution and whole brains were collected for immunofluorescence staining. For those from MPTP model, whole brain was harvested and sagittally divided into two equal parts on ice for subsequent immunofluorescence and QPCR analysis. SN, striatum, and hippocampus were dissected immediately after brain tissues were harvested. Blood samples were treated with erythrocyte lysis buffer (79217, QIAgen) on ice and centrifuged to collect white blood cells. All samples were homogenized in TRIzol reagent (15596-026, Life Technologies) for RNA extraction.

### Immunofluorescence, confocal microscopy, and image analysis

Tissues for immunofluorescence were fixed in 4% PFA solution for 24 h and then immersed in 30% sucrose for 24 h twice. Processed brain tissue was coronal sectioned at a thickness of 30 μm in four series (8–10 slices per series). Each series was incubated with primary antibodies against TH (1:500, Millipore AB152), DAT (1:100, Millipore MAB369), GFAP (1:300, DAKO Z0334), or Iba1 (1:500, Wako 019-19741), respectively, at 4 ℃ overnight, followed by secondary antibodies incubation at room temperature for 2 h. Images were obtained by confocal microscope (NiE-A1 plus, Nikon) or fluorescent microscope (E80i, Nikon) and processed by ImageJ (1.49c). The number of TH-positive cells was quantified within area of compact part of SN. The density of DAT signal was quantified by sampling 5 repeated 50 × 50 pixel areas within striatum. All quantification was single-blinded.

### Participants for HIP2 mRNA validation

Patients and controls were recruited from 4 tertiary teaching hospitals in Shanghai (Huashan Hospital, Ruijin Hospital, Zhongshan Hospital, and Shanghai First People’s Hospital) with ethical committee and institutional reviewing board approval from each hospital, and written informed consent from all participants. All PD patients were assessed clinically by at least two experienced neurologists based on the UK PD brain bank criteria and classified according to Hoehn and Yahr staging system. All PD participants received levodopa-related medical treatment except for de novo patients and levodopa equivalent daily dose (LEDD) was calculated according to previous study^35^. None of the controls suffered from neurodegenerative diseases.

In total, 211 PD patients and 91 age-matched healthy controls were recruited and assigned into 2 test sets. 52 controls and 45 patients were included in set 1, and 39 controls, 43 de novo patients, and 123 medicated patients were included in set 2. Detailed demographic information for all participants is listed in Supplementary S4. The result of HIP2 mRNA validation is presented in Supplementary S3A-D.

### Participants with instructed Tai Chi exercises and evaluation

Twenty PD patients from age of 50 to 80 were recruited to participate in the Tai Chi study for 12 months. All these patients were assessed by experienced neurologists at the Ruijin Hospital and were in Hoehn and Yahr stages I–III at the time of enrollment. All subjects were non-demented with mini-mental state examination (MMSE) > 24, without participation in any routine exercises in the last 2 years, and self-sufficient. The participants were on stable medication treatment for at least 3 months and were instructed not to change treatment plan during the current study. The exclusion criteria include any of the following: participation of any other clinical studies or exercises, falling within the last 6 months, muscle skeletal abnormalities, cardiovascular diseases, cerebral trauma, cerebral vascular diseases, heart surgery, or psychiatric and other neurological disorders.

These patients were given twice a week, 2-h long instructed session of simplified Tai Chi by professional coaches and were encouraged to practice at home. The blood samples were collected, and the clinical assessments were performed every 6 months. The HIP2 mRNA analysis and clinical assessment were performed by two independent teams double-blindly. Detailed demographic information for these participants is listed in Supplementary S5. The result is presented in Fig. 6c.

### Blood samples collection and RNA isolation

Venous blood samples were collected in BD vacutainer EDTA tube and either used for RNA extraction within 2 h or stored at −80 ℃ for later use. The total RNA from blood samples were isolated using RiboPure™-Blood Kit (AM1928, Life Technologies) and processed according to manufacturer’s protocol.

### QPCR analysis

For both RNA samples of animal tissue and human blood, 2 μg of total RNA was reverse transcribed into cDNA using a mixture of oligo (dT) 18 primer (3806, TaKaRa), dNTP mixture (4019, TaKaRa), Recombinant RNase Inhibitor (2313A, TaKaRa) and Reverse Transcriptase M-MLV(RNase H-) (2641A, TaKaRa) according to the manufacturer’s instructions. 10 ng of total cDNA was then mixed with iQ^TM^ SYBR Green Supermix (170-8882AP, Bio-Rad) and primers of target genes. QPCR was performed on CFX connect Real-Time system (Bio-Rad Laboratories) with a program as follows: first, denaturation for 2 min at 94 ℃, followed by 40 cycles of denaturation for 15 s at 94 ℃, annealing for 20 s at 60 ℃, and extension for 15 s at 72 ℃.

The sequence of primers can be found in Supplementary S6. The specificity of primers was confirmed by NCBI Primer-BLAST, melting curve analysis, and DNA electrophoresis (data not shown). The possibility of non-specific amplification of all primers was ruled out by DNA sequencing of QPCR product. For QPCR data analysis, HIP2 expression level was firstly normalized to GAPDH and presented as the fold change relative to mean value of saline group (Fig. 5c–f), healthy control (Supplementary S3A&C), or beginning level of HIP2 (Fig. 6c) following the instruction of previous study^36^. Additional reference genes including HPRT1, RPL13A, SDHA, PPIA, and GUSB were used to validate the effectiveness of GAPDH as the main reference gene (Supplementary S7A-D). The selection of these reference genes was based on prior evaluation and their expression in the brain and blood^37–43^. We have further validated that the expression of these housekeeping/reference genes was not altered in the PD samples or in MPTP-treated mice from our study and in prior studies (Supplementary S7E–L). To avoid discrepancy between each QPCR run, same quality control samples were amplified in each run in either mouse or human samples.

### Statistics analysis

Statistical analyses were performed using GraphPad Prism (version 5.0, GraphPad Software). Unpaired Student’s *t*-test was used for Gaussian distributed statistics (Figs. 1e, 2b–e, 3i, 4c, e, 5b–f, S2A–E, S3A, and S7A–D). Due to the inconsistency of participants (2 patients dropped out in 12 months), paired *t*-test was used to compare matched groups in Tai Chi exercise (Fig. 6a, c). One-way analysis of variance (ANOVA) with Tukey post-test was applied after Fisher’s exact test was examined (Fig. S3C). Two-way ANOVA followed by Bonferroni’s post-hoc test was applied in Figs. 3a–h,  4b, and S7E–L.

## Electronic supplementary material

Supplementary S1

Supplementary S2

Supplementary S3

Supplementary S4

Supplementary S5

Supplementary S6

Supplementary S7
