# Supplementary material for: Reduction of HIP2 expression causes motor function impairment and increased vulnerability to dopaminergic degeneration in Parkinson’s disease models
Source: Cell Death Dis. 2018 Oct 3;9(10):1020. doi: 10.1038/s41419-018-1066-z (PMC6170399; doi:10.1038/s41419-018-1066-z)
Supplement: Supplementary file 1 — Supplementary S1 [file 41419_2018_1066_MOESM1_ESM.pdf]

A

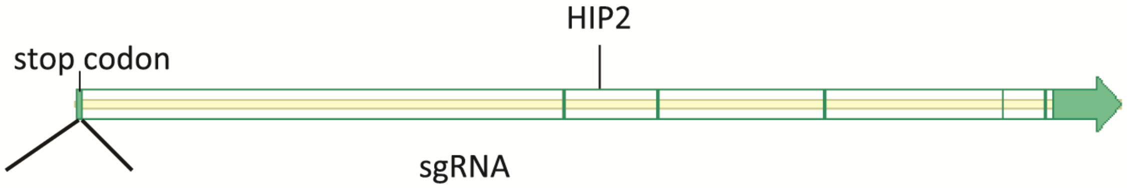

Targeted sequence: 5'-...ATGGCCAACATCGCGGTGCAGCGAATCAAGCGG...-3'

Repair oligo: 5'-...ATCGCGGTGCAGCGAATCAGGCGGGAGTTCAAGGAGGT...-3'

5'-homology arm(50bp) 3'-homology arm(50bp)

B

Project	Strain	Injected embryos	Surrogates	Survival/ Total newborns	Single clone examination			
					WT	PM	PM+Del.	PM+Ins.
HIP2 K10	C57BL/6	220	7	16/28	11/16	3/16 (#20, #21, #26)	1/16 (#16)	1/16 (#27)

C

F0	Mutation sequence	Deletion/Insertion	Stop codon site	F1 offspring
#16	ATCGCGGTGCAGCG----AAGCGG	Deletion (-4bp)	15aa	No offspring
#27	ATCGCGGTGCAGCGAATCGGAAGCGG	Insertion (-2bp)	17aa	2 heterozygotes

D

No.	Sequence	Chr.	Position	Gene	Mismatches	Mouse	
						#16	#27
OT-1	TTGCTGGGCAGCGAATCAAA GGG	chr 12	104914732	None	4(2,5,7,20)	--	--
OT-2	TCACTGTGCAGGCAATCAAG TGG	chr 2	51419286	None	4(3,5,12,13)	--	--
OT-3	TCGCGGTTCTGCAAATCAAT AGG	chr 15	91795927	None	4(8,10,13,20)	--	--
OT-4	TAGCGGTGTAGAGAATAAAG GGG	chr 7	143394289	None	4(2,9,12,17)	--	--
OT-5	TCCCTGTGCAGCGAACCAAA AGG	chr 2	128419623	None	4(3,5,16,20)	--	--
OT-6	TAGAGGTGCAGCTTATCAAG TGG	chr 9	110130083	None	4(2,4,13,14)	--	--
OT-7	TCGGGGTGCAGCGACTCAGC CGG	chr 9	64371099	None	4(4,15,19,20)	--	--
OT-8	TCGCTCTGCAGCGCATCCAG GGG	chr 17	67902184	None	4(5,6,14,18)	--	--

S1 The generation of HIP2 KO mice

(A) Schematic representation of sgRNA-targeting sequence of HIP2 and its repair oligo. (B) Statistics of natal HIP2 K10R mice, 2 out of 16 newborns carried deletion or insertion leading to frame shift and early stop codon. (C) Detailed information about HIP2 knockout mosaic lines #16 and #27. Note only #27 generated offspring at F1 generation. (D) Evaluation of potential off-target effects result in #16 and #27. Algorithm-predicted off-target effects from sgRNA sequences were examined and no unintended gene editing was found.
