# Supplementary material for: Reduction of HIP2 expression causes motor function impairment and increased vulnerability to dopaminergic degeneration in Parkinson’s disease models
Source: Cell Death Dis. 2018 Oct 3;9(10):1020. doi: 10.1038/s41419-018-1066-z (PMC6170399; doi:10.1038/s41419-018-1066-z)
Supplement: Supplementary file 2 — Supplementary S2 [file 41419_2018_1066_MOESM2_ESM.pdf]

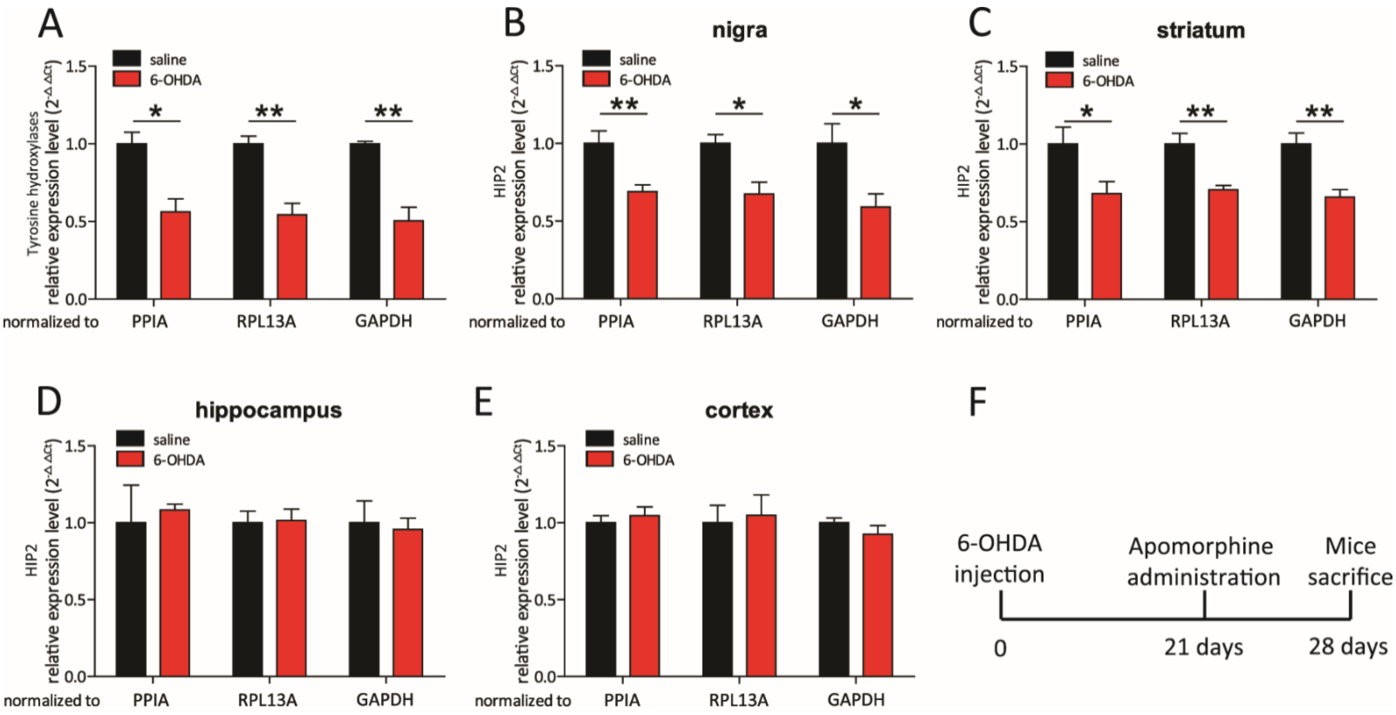

S2 The HIP2 mRNA is decreased in 6-OHDA-treated mice

(A) Validation of 6-OHDA efficacy by reduced TH mRNA level in the substantia nigra of injected hemisphere. TH mRNA expression was normalized to each indicated reference gene (PPIA, RPL13A or GAPDH). (N = 3 in saline group and n=6 in 6-OHDA group, *: $P<0.05$, **: $P<0.01$ by unpaired t-test). (B-E) HIP2 mRNA expression was significantly decreased in the substantia nigra (B) and striatum (C), but not in the hippocampus (D) or cortex (E). HIP2 mRNA expression was normalized to each indicated reference gene (N = 3 in saline group and n=6 in 6-OHDA group, *: $P<0.05$, **: $P<0.01$ by unpaired t-test). (F) Timeline of behavioral and pathologic assessment of 6-OHDA mice. After recovery from operation, mice were administrated with apomorphine to induce rotation behavior at the 21$^{st.}$ day, and were sacrificed for pathologic assessment at the 28$^{th.}$ day.
