# Supplementary material for: Reduction of HIP2 expression causes motor function impairment and increased vulnerability to dopaminergic degeneration in Parkinson’s disease models
Source: Cell Death Dis. 2018 Oct 3;9(10):1020. doi: 10.1038/s41419-018-1066-z (PMC6170399; doi:10.1038/s41419-018-1066-z)
Supplement: Supplementary file 3 — Supplementary S3 [file 41419_2018_1066_MOESM3_ESM.pdf]

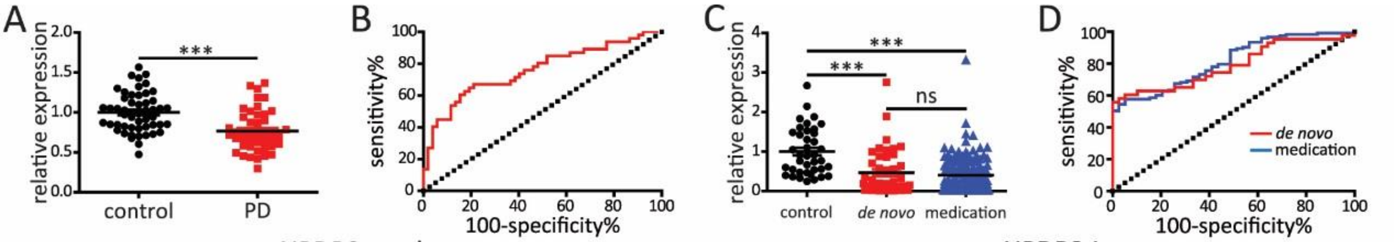

E UPDRS-total

6 months

HIP2 \ UPDRS	Increased	No change	Decreased
Increased	2	0	15
No change	0	0	2
Decreased	1	0	0
Consistency: 0.8			

12 months

HIP2 \ UPDRS	Increased	No change	Decreased
Increased	0	1	12
No change	0	0	1
Decreased	1	0	3
Consistency: 0.72			

F UPDRS-I

6 months

HIP2 \ UPDRS	Increased	No change	Decreased
Increased	1	5	11
No change	0	0	2
Decreased	0	1	0
Consistency: 0.55			

12 months

HIP2 \ UPDRS	Increased	No change	Decreased
Increased	1	2	10
No change	0	1	0
Decreased	2	0	2
Consistency: 0.72			

G UPDRS-II

6 months

HIP2 \ UPDRS	Increased	No change	Decreased
Increased	1	3	13
No change	0	0	2
Decreased	0	1	0
Consistency: 0.65			

12 months

HIP2 \ UPDRS	Increased	No change	Decreased
Increased	1	3	9
No change	0	0	1
Decreased	1	0	3
Consistency: 0.56			

H UPDRS-III

6 months

HIP2 \ UPDRS	Increased	No change	Decreased
Increased	2	1	14
No change	0	1	1
Decreased	1	0	0
Consistency: 0.8			

12 months

HIP2 \ UPDRS	Increased	No change	Decreased
Increased	0	1	12
No change	0	0	1
Decreased	0	1	3
Consistency: 0.67			

I UPDRS-IV

6 months

HIP2 \ UPDRS	Increased	No change	Decreased
Increased	0	15	2
No change	1	1	0
Decreased	0	1	0
Consistency: 0.15			

12 months

HIP2 \ UPDRS	Increased	No change	Decreased
Increased	0	11	2
No change	1	0	0
Decreased	0	4	0
Consistency: 0.11			

S3 The HIP2 mRNA is decreased in the blood of PD patients and reversed after Tai Chi training.

(A) The HIP2 mRNA level in blood samples from test set 1 (***: $P<0.001$ by unpaired t-test). (B) Receiver operating characteristics (ROC) curve of HIP2 mRNA from PD vs. control, with an area under the curve (AUC) of 0.77. (C) The HIP2 mRNA level in blood samples from test set 2 (ns: $P>0.05$, ***: $P<0.001$ by one-way ANOVA with Tukey post-test). (D) ROC curves of HIP2 mRNA from *de novo* and medication vs. control, with AUC of 0.79 for the *de novo* and 0.81 for the medication group. (A-D)Detailed demographic information of 2 test sets was listed in Supplementary S3. (E-I)Detail information of the change of HIP2 level with that of UPDRS scores in PD patients undergoing Tai Chi exercises. Patients that showed increased HIP2 mRNA level along with decreased UPDRS score or decreased HIP2 level with increased UPDRS score, or those shown less than 10% change in both HIP2 level and UPDRS score, were considered to be “consistent cases”. Other patients were classified as “inconsistent”. Data are presented as mean ± SEM.
