# Supplementary material for: Reduction of HIP2 expression causes motor function impairment and increased vulnerability to dopaminergic degeneration in Parkinson’s disease models
Source: Cell Death Dis. 2018 Oct 3;9(10):1020. doi: 10.1038/s41419-018-1066-z (PMC6170399; doi:10.1038/s41419-018-1066-z)
Supplement: Supplementary file 4 — Supplementary S4 [file 41419_2018_1066_MOESM4_ESM.pdf]

Test set	Group	n(M/F)	Age (years)	H-Y stage	UPDRS-III score	LEDD(mg)
1	control	52(24/28)	58.6(14.7)	0	0	0
	PD	45(22/23)	61.7(7.6)	2.1(0.7)	28.7(12.6)	413.0(332.8)
2	control	39(22/17)	62.2(7.5)	0	0	0
	de novo	43(24/19)	62.2(5.9)	1.6(0.7)	22.3(9.7)	0
	medication	123(63/60)	62.8(6.7)	2.3 (1)	28.9 (14.6)	386.5(269.5)

S4 Table Demographics of Participants for HIP2 mRNA validation

M/F: male/female. H-Y stage: Hoehn & Yahr stage. UPDRS: unified Parkinson's disease rating scale. LEDD: levodopa equivalent daily dose. Data are shown as Mean (SD).
