# Supplementary material for: Reduction of HIP2 expression causes motor function impairment and increased vulnerability to dopaminergic degeneration in Parkinson’s disease models
Source: Cell Death Dis. 2018 Oct 3;9(10):1020. doi: 10.1038/s41419-018-1066-z (PMC6170399; doi:10.1038/s41419-018-1066-z)
Supplement: Supplementary file 5 — Supplementary S5 [file 41419_2018_1066_MOESM5_ESM.pdf]

N (M/F)	Age	Disease duration	H-Y stage	UPDRS-III score	BBS	TUG	LEDD
20 (10/10)	63.1 (5.3)	6.2 (4.5)	1.8 (0.7)	25.2 (14.1)	49.7 (3.5)	11.8 (2.6)	314.4 (186.5)

S5 Table Demographics of PD patients with Tai Chi exercises.

M/F: male/female. H-Y stage: Hoehn & Yahr stage. UPDRS: unified Parkinson's disease rating scale. BBS: Berg Balance Scale. TUG: Time Up and Go test. LEDD: levodopa equivalent daily dose. Data are shown as Mean (SD).
