# Supplementary material for: Reduction of HIP2 expression causes motor function impairment and increased vulnerability to dopaminergic degeneration in Parkinson’s disease models
Source: Cell Death Dis. 2018 Oct 3;9(10):1020. doi: 10.1038/s41419-018-1066-z (PMC6170399; doi:10.1038/s41419-018-1066-z)
Supplement: Supplementary file 6 — Supplementary S6 [file 41419_2018_1066_MOESM6_ESM.pdf]

Genes	5'-3' sequences	Primer site	Amplicon size(bp)
Human GAPDH	AAGATCATCAGCAATGCCTCC	509-529	107
NM_002046	GCATGGACTGTGGTCATGAG	615-596	
Human HPRT1	GACCAGTCAACAGGGGACAT	489-508	195
NM_000194	AACACTTCGTGGGGTCCTTTTC	683-662	
Human RPL13A	AAAAGCGGATGGTGGTTCCT	420-439	118
NM_012423	GCTGTCACTGCCTGGTACTT	537-518	
Human PPIA	ACGTGGTATAAAAGGGGCGG	9-28	163
NM_021130	TGTCTGCAAACAGCTCAAAGG	151-171	
Human SDHA	AAACTCGCTCTTGGACCTGG	1495-1514	111
NM_004168	TCTTCCCCAGCGTTTGGTTT	1586-1605	
Human HIP2	CATACCCATTTAATCCCCCT	478-497	227
NM_005339	CATTTCGGGATTTTGTTTGT	704-685	
Mouse GAPDH	AGGTCGGTGTGAACGGATTTG	100-120	123
NM_001289726	TGTAGACCATGTAGTTGAGGTCA	222-200	
Mouse PPIA	TATCTGCACTGCCAAGACTGAATG	381–404	127
NM_008907	CTTCTTGCTGGTCTTGCCATTCC	507–485	
Mouse RPL13A	TGTGGCCAAGCAGGTACTTC	97-116	133
NM_009438	GGGGTTGGTATTCATCCGCT	229-210	
Mouse GUSB	ATCAACAACACACTGACCCCT	744-764	232
NM_010368	AAATCCAGTAGGTCACCAGCC	955-975	
Mouse HPRT	CTCATGGACTGATTATGGACAGGAC	259-283	123
NM_013556	GCAGGTCAGCAAAGAACTTATAGCC	357-381	

Mouse HIP2	TTCCGTCACAGGGGCTATT	494-512	227
NM_016786	CTGGACTAGAAACTGGTGCT	720-701	
Mouse TH	GTCAGAGGAGCCCGAGGTC	1246-1264	150
NM_009377	CGCTGGATACGAGAGGCATAG	1375-1395	

S6 QPCR primer sequences for human and mouse samples
