# Supplementary material for: Reduction of HIP2 expression causes motor function impairment and increased vulnerability to dopaminergic degeneration in Parkinson’s disease models
Source: Cell Death Dis. 2018 Oct 3;9(10):1020. doi: 10.1038/s41419-018-1066-z (PMC6170399; doi:10.1038/s41419-018-1066-z)
Supplement: Supplementary file 7 — Supplementary S7 [file 41419_2018_1066_MOESM7_ESM.pdf]

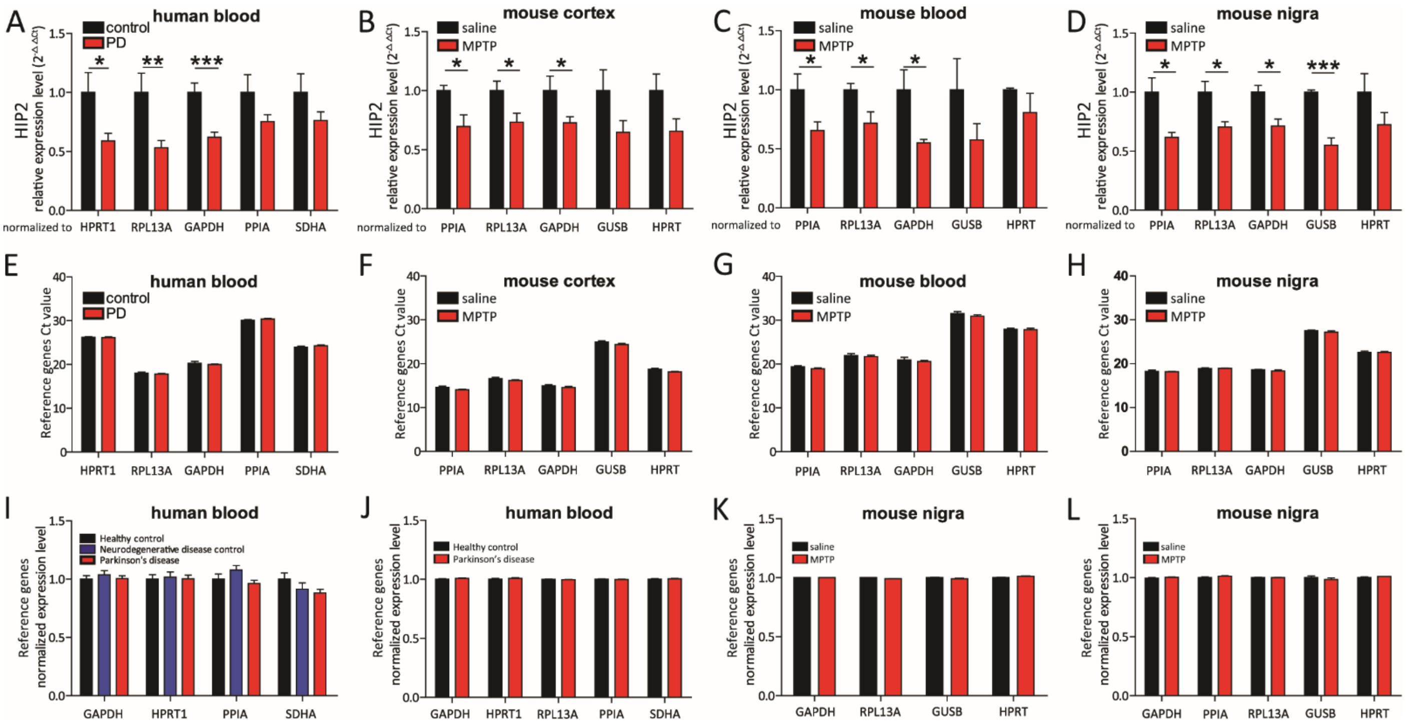

S7 Quantification of HIP2 expression using multiple reference genes.

(A) Decreased HIP2 expression was observed using 5 different reference genes in human blood samples (N = 10 in both control and PD groups, *: *P*<0.05, **: *P*<0.01, ***: *P*<0.001 by unpaired t-test). (B-D) Decreased HIP2 expression was observed when normalized to 5 different reference genes in mouse cortex (B, N=4 in saline group and n=6 in MPTP group), blood (C) and substantia nigra (D) (saline group=4 and MPTP group=5 in C&D, *: P<0.05, ***: P<0.001 by unpaired t-test). (E) Stable Ct value of various reference genes in the peripheral blood from the control and PD patients. (F-H) Stable Ct value of various reference genes in mouse cortex samples (F), blood samples (G) and substantia nigra samples (H) from control or MPTP treated mice. (I-J) The expression of reference genes in peripheral blood retrieved from 2 GEO datasets (I, GSE6613 J, GSE72267) was not affected by disease state. (K-L) The expression of reference genes in the substantia nigra retrieved from 2 GEO datasets (K, GSE4788 L, GSE7707) was not altered by MPTP treatment. Values represent mean ± SEM.
